# Rising Health Expenditure Due to Non-Communicable Diseases in India: An Outlook

**DOI:** 10.3389/fpubh.2016.00268

**Published:** 2016-11-29

**Authors:** Debasis Barik, Perianayagam Arokiasamy

**Affiliations:** ^1^National Council of Applied Economic Research, New Delhi, Delhi, India; ^2^International Institute for Population Sciences, Mumbai, Maharashtra, India

**Keywords:** non-communicable diseases, India, health care, South Asia, burden

## Abstract

With ongoing demographic transition, epidemiological transition has been emerged as a growing concern in India. The share of non-communicable disease in total disease burden has increased from 31% in 1990 to 45% in 2010. This paper seeks to explore the health scenario of India in the wake of the growing pace of non-communicable diseases such as diabetes and hypertension among Indian population using data from health and morbidity survey of the National Sample Survey Organisation (2004) and notifies about the resource needed to tackle this growing health risk. Given the share of private players (70%) in Indian health system, results indicate a higher private expenditure, mostly out-of-pocket expense, on account of non-communicable diseases. A timely look into the matter may tackle a more dreadful situation in near future.

## Background

Through the early and mid-phase of India’s epidemiological transition, the age pattern of morbidity was overwhelmed with infectious diseases (1). In the recent two decades, the prevalence of non-communicable diseases has escalated immensely in India (2). India’s swift progress through demographic and epidemiological transition has resulted into a bigger challenge of dual burden of communicable and non-communicable diseases (3, 4). With the share of older cohorts increasing relative to that of younger cohorts, infectious and nutritional disorders are being replaced by chronic, degenerative, and mental illnesses as the leading causes of morbidity and mortality (5, 6). The resulting concern is not just epidemiological but also economic. Non-communicable diseases, which could cause long-standing disabilities, have direct economic consequences at households and community level, both through the expenses on health care, which divert other expenditure, and also on levels of income through reduced labor productivity (7, 8).

Expenditure on health is highly lopsided across the globe, where the developed countries spend the most on health per person (9). The Organisation for Economic Co-operation and Development (OECD) countries accounted for less than 20% of the world’s population but were responsible for almost 90% of the world’s health spending (10). Health expenditure, both in terms of percentage of gross domestic product (GDP) spent on health and per capita health expenditure, is much higher in the developed countries than developing countries. Unlike in economic history of Colonial Age, in recent decades, global share of health spending is undergoing gradual but constant change in favor of developing Third World nations (11). Such inevitable and deep changes are led by top BRICS^1^ emerging markets (12). In contrast, the ratio of public to private health expenditure is extremely low in India. Further, all the private expenditure in India (as in some other countries) almost exclusively represent household’s out-of-pocket health expenses. According to World Health Organization (WHO) (13), private share on total health-care spending in India is as high as 70%, only next to some poor African countries. In India, 86% of all private expenditure and 72% of total expenditure are out-of-pocket. These high out-of-pocket spending on health often takes a higher toll on the poor than the rich (14). Poor people in India spend about 15% of their monthly household income for medical treatment (15).

South Asian countries are going through a rapid demographic transition, which is often accompanied by epidemiological transition. In this phase, while communicable diseases are still pandemic, non-communicable diseases, such as cardiovascular disease, diabetes, respiratory diseases, and cancer, are picking pace. Ghaffar et al. (16) pointed out the growing risk of these diseases among South Asian population. The non-communicable disease in this region has some special feature in addition to those commonly available in the developing countries. The metabolic syndrome is very common among urban Indians (17, 18). The high prevalence of glucose intolerance and the distinctive dyslipidaemic pattern of reduced concentrations of high density lipoprotein (HDL) cholesterol and high concentrations of triglycerides characterize the metabolic profile, and abdominal obesity characterizes the phenotype of the urban adult in India. Impaired maternal and fetal nutrition, resulting into low birth weight poses a greater risk of cardiovascular disease in South Asia. While use of tobacco products increases the risk of cancer in the region, indoor air pollution mostly due to burning wood fuel contributes to chronic obstructive airway disease (16). Barik et al. (19), in their very recent article, has argued that richer Indian adults are at greater risk of developing non-communicable diseases such as diabetes, heart disease, and high blood pressure than their poorer counterparts. The report of the Global Burden of Disease reported that the share of non-communicable disease on the disease burden has increased from 31% in 1990 to 45% in 2010 (20).

Following the demographic transition, developed western countries have witnessed an epidemiological transition where the share of non-communicable diseases increased in the total disease burden. The non-communicable diseases require a very different health set up than which is required for communicable diseases. In the present paper, we have tried to project the future health scenario of India in the wake of demographic transition and associated health transition. We have estimated the likely scenario of share of communicable and non-communicable diseases among Indians and required economic resource for treatment of these maladies. Various countries have developed their National Health Accounts (NHA) estimates to plan their health budget according to the nature of diseases. India does not have such a comprehensive NHA estimate. This present study may throw some light to plan health budget according to the need of the hour and also for future.

## Data

We have used the Morbidity and Health care Survey (2004) 60th round, carried out by the National Sample Survey Organization (NSSO) under the Ministry of Statistics and Programme Implementation, Government of India as the main data source for the analysis. The survey was canvassed between January to June, 2004 across the whole of the Indian Union except (i) Leh (Ladakh) and Kargil districts of Jammu and Kashmir, (ii) interior villages of Nagaland situated beyond 5 km of the bus route, and (iii) villages in Andaman and Nicobar Islands which remain inaccessible throughout the year. It covered 73,868 households of which 47,302 were from rural areas and 26,566 from urban areas. Prevalence of various morbidities and utilization of health-care services from public and private sources, together with the expenditure incurred by the households in this process, have been collected through this survey. The survey collected information if a person was ailing during the last 15 days prior to the date of survey. The individual sample size was 383,338; of which 195,712 were males and 187,626 females.

The data collected can be summarized in three categories:

- The first category comprises the inpatient health care received by the household members as hospitalized cases during last 365 days. The expenses incurred during the reference period for treatment (as an inpatient of a hospital) of such ailments and details of the health-care finance have been recorded here.
- Irrespective of the hospitalization status, the second category documents all such ailments for which the patients were treated during the 15 days prior to the survey.
- The third category includes all ailments suffered during 15 days prior to the date of survey for which no medical treatment received.

## Methodology

The classification of communicable and non-communicable diseases followed the classification adopted by WHO in Global Burden of Disease Studies, 2004 (Table A1 in Appendix). The prevalence of any ailment or morbidity has been defined as the number of persons ailing within the reference period per 1,000 persons exposed to the risk of the ailment from the same group of population.

The expected change in the share of each category of morbidities has been estimated up to year 2051. The main assumption behind this projection was that the prevalence rate of each morbidity category would remain unchanged among all the age groups over time. This is a very strong assumption, but we did not have a dataset which allows us to examine disease progression in Indian context. However, this demographic projection would help us to foresee the future of Indian epidemiology in the course of demographic transition, the process already started. Utilization of hospitalization services over the 15-day reference period was multiplied by 24.33 (i.e., 365/15) to obtain an annualized counterpart of hospitalizations (after netting out any hospitalizations reported in the 15-day reference period).

The yearly cost of treatment for communicable and non-communicable diseases has been estimated based on per unit cost of treatment for inpatient and outpatient, derived from the NSSO unit-level data. Age-specific hospital days have been estimated for each disease type. The average cost of treatment per stay as an inpatient and average duration of stay were estimated for each disease type to obtain per day average cost of treatment as inpatients, and multiplying it with the estimated hospital days provides the annual cost of treatment as an inpatient. The annual out-of-pocket expenditure as outpatient has also been estimated following the similar procedure, where out-of-pocket expenditure implies to the expenditure a household bears after deducting the reimbursement from various health-care financing sources. We reached the annual cost of treatment as an outpatient by multiplying the average cost of treatment with total number of outpatient visit. Age-specific inpatient and outpatient visit has been assumed to capture the effect of the increasing prevalence of non-communicable diseases beyond age 30 years.

Household level out-of-pocket expenses are the major source of health-care finance in India. Since the presence of health insurance in India is yet to feel, the rest of the health expenditure are met by the government *via* its provision of free or subsidized public facilities (21). Thus, we will focus primarily on only these two sources of health-care financing, referring to other funding sources when appropriate. Public subsidies for health have been estimated as the difference between total expenditure incurred on private facilities compared to public facilities. The entire analysis has been carried out using appropriate sample weight available in the dataset. SPSS 18 PASW software was used to analyze the data.

## Results

### Age-Specific Prevalence of Communicable and Non-Communicable Diseases in India, 2004

**Figure 1:**
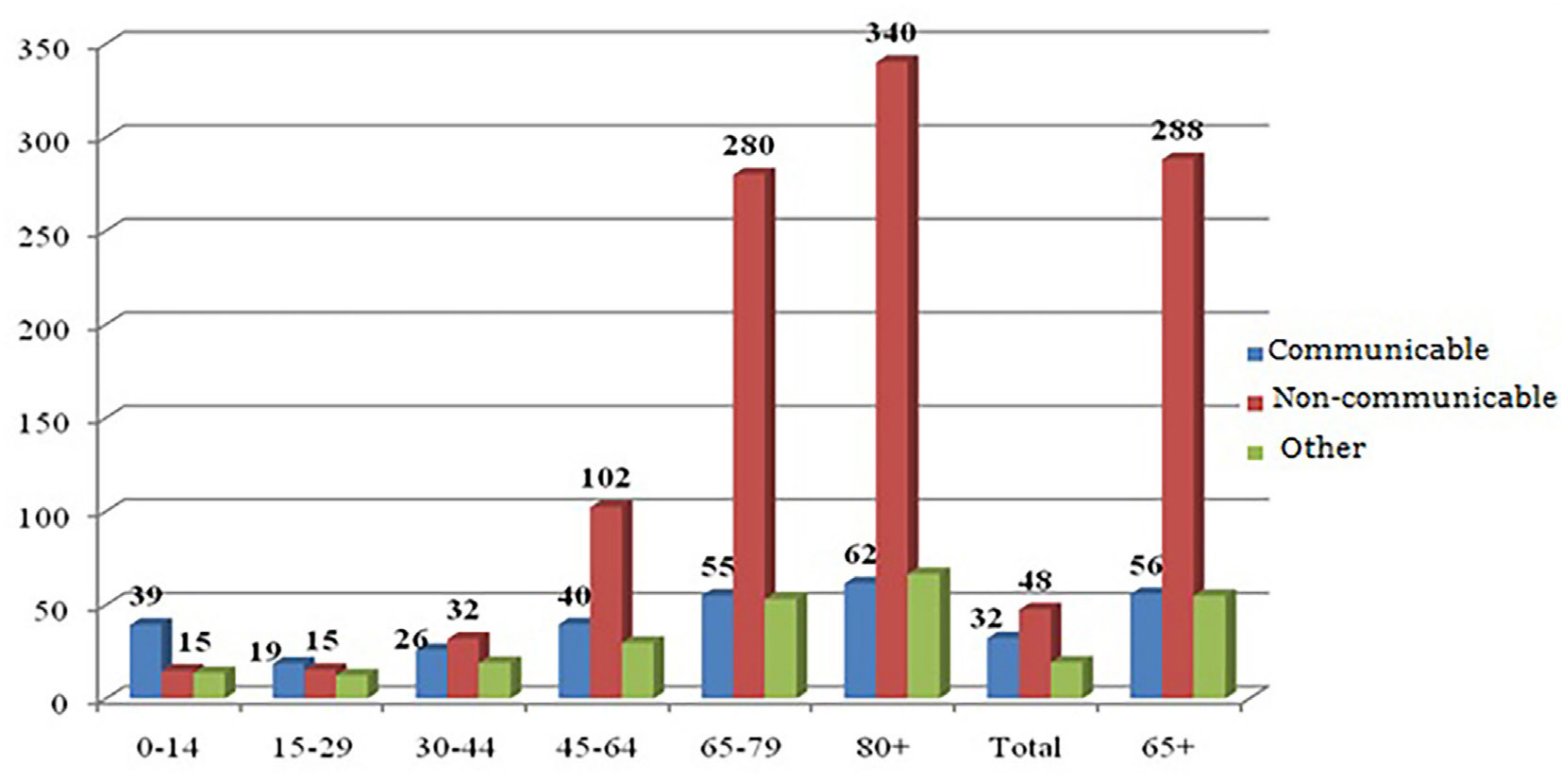
**Age-specific prevalence of communicable, non-communicable, and other types of diseases in India, 2004**. Source: authors’ own calculation from NSSO 60th round unit-level data.

In 2004, the prevalence of the communicable and non-communicable diseases reported in the morbidity and health survey was 32 and 48, respectively, per 1,000 Indian population (Figure 1). We see a dominance of communicable diseases in the very early stages of life, but with the advancement of the age, the prevalence of non-communicable disease rises manifold. Though the prevalence of non-communicable diseases rises continuously after age 30 years, it becomes dominant mainly beyond age 45 years and increases gradually with the increase in age. The prevalence of either communicable or non-communicable disease is lowest among the population of 15–29 years age group. With the increase in the age, the prevalence of both communicable and non-communicable disease increases, but the latter increased at a much faster rate. The prevalence of communicable diseases has been recorded as 56 and 62 among the old (65+ years) and oldest old (80+ years) people, respectively. In contrast, the prevalence of non-communicable diseases for the same age group was much higher, counting 288 and 340 respectively.

### Non-Communicable Diseases Will Take Even a Higher Share among Indian Population by 2051

**Figure 2:**
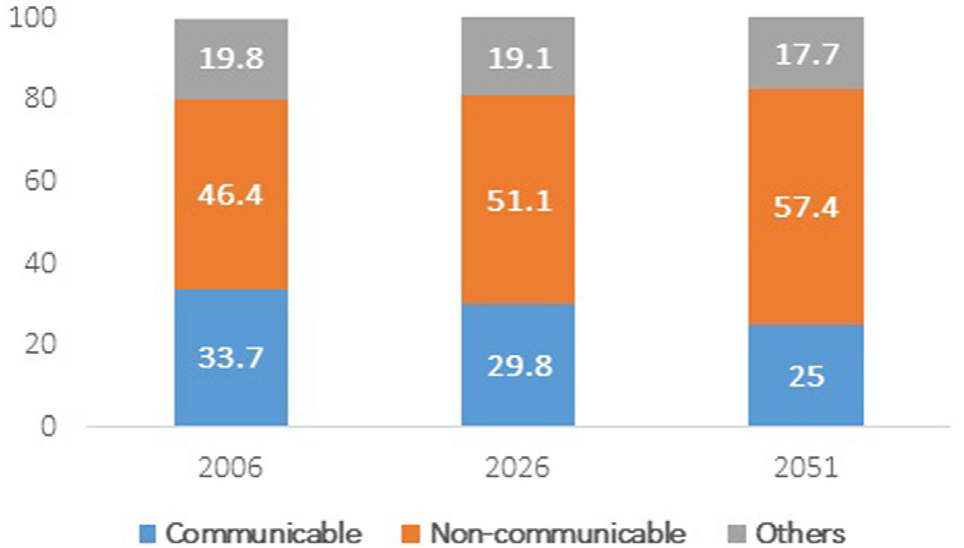
**Projected share of communicable, non-communicable and other types of diseases among total population 2006–2051**. Source: authors’ own calculation from NSS 60th round unit-level data.

In India, about half of the disease burden was shared by non-communicable diseases (46.4%), such as diabetes, high blood pressure, cardiac condition, whereas the share of communicable diseases, such as tuberculosis and malaria, was 34% (Figure 2). The share of communicable diseases was likely to reduce to one-fourth by 2051. At the same time, the share of non-communicable diseases has been projected to increase to 57.4%. A minimal decline will be observed in the share of other health condition during the same period. This shift in share will be observed mainly due to the shift in age distribution of population toward elderly hood.

### Health Spending on Non-Communicable Diseases and Government Subsidies

There were 75 million hospitalization cases during January to June, 2004, out of which 35 million were in government health facilities and 40 million in private facilities (Table 1). We did not find any remarkable difference in hospitalization due to communicable and non-communicable diseases. Hospitalization in government facilities for communicable diseases was relatively higher than for non-communicable diseases. People usually rush to private facilities for hospitalization in case of non-communicable diseases. Beyond age 45 years, the volume of hospitalized persons due to non-communicable diseases was higher than communicable diseases in both government and private facilities. Again, the average duration of stay as an inpatient was much higher for non-communicable diseases than for communicable diseases (Table 2). Once hospitalized, the average duration of stay in government facilities was higher than private facilities. The number of hospital days was higher for non-communicable diseases than communicable diseases in both government as well as private facilities.

**Table 1:** **Total number of person hospitalized, average duration of stay as inpatient, and total number of hospital days in government and private sector, 2004**.

	**Hospitalization – government (in million)**					**Average duration of stay – government (days)**					**Hospital days – government (million)**				
	**0–34**	**35–44**	**45–64**	**65+**	**Total**	**0–34**	**35–44**	**45–64**	**65+**	**Total**	**0–34**	**35–44**	**45–64**	**65+**	**Total**
CD	7.81	2.39	2.98	0.85	14.19	7.39	9.49	11.26	9.6	8.81	57.7	22.7	33.6	8.2	122.2
NCD	3.84	1.79	3.57	1.90	11.69	10.83	15.34	12.75	10.29	11.96	41.5	27.4	45.6	19.5	134.0
Others	5.00	1.80	1.95	0.59	9.44	10.19	14.62	12.65	12.08	11.64	51.0	26.2	24.7	7.2	109.1
Total	16.65	5.98	8.51	3.34	35.32	9.18	13.08	12.24	10.4	10.72	152.8	78.2	104.1	34.8	369.9

	**Hospitalization – private (in million)**					**Average duration of stay – private (days)**					**Hospital days – private (million)**				

CD	7.30	1.91	2.53	0.89	12.79	5.97	6.4	7.73	8.34	6.65	43.6	12.2	19.5	7.4	82.8
NCD	5.15	2.50	4.68	2.35	15.43	8.39	8.76	9.05	8.5	8.68	43.2	21.9	42.4	20.0	127.5
Others	6.00	2.10	2.52	0.91	11.72	8.7	9.92	9.92	10.15	9.33	52.2	20.9	25.0	9.3	107.3
Total	18.46	6.52	9.72	4.16	39.94	7.55	8.36	8.91	8.83	8.21	139.4	54.5	86.6	36.7	317.2

*Source: authors’ own calculation from NSS 60th round unit-level data*.

**Table 2:** **Average cost per stay and average cost of hospitalization per day by source of providers, 2004**.

	Average cost per stay as inpatient (INR)					Per day average cost of hospitalization (INR)				
	0–34	35–44	45–64	65+	Total	0–34	35–44	45–64	65+	Total
CD	2,815	3,079	3,369	3,346	3,034	381	324	299	349	344
NCD	5,643	8,522	8,030	5,918	6,876	521	556	630	575	575
Others	4,463	5,701	5,748	5,321	5,014	438	390	454	440	431
Govt.	4,071	5,658	5,963	5,120	4,935	444	433	487	492	460
CD	5,763	10,522	9,877	12,347	8,015	965	1,644	1,278	1,480	1,205
NCD	10,262	16,263	17,799	14,244	14,377	1,223	1,857	1,967	1,676	1,656
Others	10,080	12,278	13,733	14,249	11,718	1,159	1,238	1,384	1,404	1,256
Pvt.	8,454	13,202	14,613	13,835	11,552	1,120	1,579	1,640	1,567	1,407

*Source: authors’ own calculation from NSS 60th round unit-level data*.

The average cost of hospitalization per stay at private facilities (INR 11,552) was much higher than in public facilities (INR 4,935) for any type of illnesses (Table 2). For non-communicable diseases, average cost per stay as an inpatient was as high as INR 14,377 in private compared to INR 6,876 in government. The average cost of treatment either as inpatient or outpatient was higher in private than in public. Each inpatient day accounted more than three times higher cost in private facilities than in public. In government facilities, average per day cost of hospitalization for communicable diseases was high among the youth (age less than 34 years) and the elderly (65 years and above). People of the prime working age (35–44 years) usually spent more on private facilities when suffered from communicable diseases.

There were nearly 1,740 million outpatient visits during 2004, out of which 380 million were in government and 1,360 million in private. Outpatient visit was higher in private facilities than government in all the age groups. Outpatient visit due to communicable diseases was highest in 0- to 34-year age group in both government and private facilities but resurged with age. Outpatient visit for non-communicable diseases increased with age in both types of health facilities (Table 3). Similar to inpatient care, per visit cost of outpatient treatment was much higher in private facilities than public facilities. Although the cost of treatment in private was higher than that of public, people rushed to private facilities when in need. This signifies the shortfall of public health systems either in reach or in belief or both in Indian context. Cost of treatment for outpatient care was also higher for non-communicable diseases than communicable diseases in both types of facilities.

**Table 3:** **Particulars of outpatient care visits, per visit total expenditure, and out-of-pocket expenditure by type of diseases and source of providers, 2004**.

	Total number of visit for outpatient care (in millions)					Per visit cost (INR) for outpatient care					Per visit OOP cost (INR) for outpatient care				
	0–34	35–44	45–64	65+	Total	0–34	35–44	45–64	65+	Total	0–34	35–44	45–64	65+	Total
CD	88.7	13.5	29.0	9.4	140.7	189.2	207.2	222.4	236.7	201.0	184.7	205.4	221.2	236.7	197.7
NCD	40.7	22.3	52.6	49.2	164.1	223.3	187.0	238.7	208.6	219.2	221.5	183.9	235.3	202.8	215.6
Others	38.9	12.8	15.5	11.8	78.7	217.7	406.0	166.1	242.5	241.4	182.0	404.1	164.8	241.7	223.1
Govt.	168.3	48.6	97.1	70.4	383.6	204.0	250.2	222.3	218.0	217.1	193.0	247.8	219.9	213.8	210.6
CD	381.7	57.5	79.1	32.9	550.9	248.8	274.1	401.8	375.6	281.3	248.5	273.2	399.4	372.6	280.5
NCD	145.3	63.6	180.0	153.2	540.2	321.3	352.6	374.7	339.5	348.2	318.8	351.7	369.1	339.0	345.4
Others	142.6	38.7	54.6	32.2	267.8	329.7	330.8	314.8	260.1	318.8	323.4	330.8	311.7	259.4	314.7
Pvt.	669.6	159.8	313.7	218.2	1,358.8	281.8	319.1	371.1	333.2	315.3	279.7	318.4	366.7	332.3	313.0

*Source: authors’ own calculation from NSS 60th round unit-level data*.

Higher cost of treatment among the elderly age group compared to younger age groups has a clearer implication on future health-care cost since the proportion of elderly has been projected to increase substantially with a rising burden of non-communicable diseases. Per visit out-of-pocket cost of treatment was similar to the total cost of treatment – indicates the failure of health insurance as a potential tool for effective health-care finance. This high out-of-pocket health spending leads to the catastrophic level of spending for health care and pushes households to poverty trap (22–24). Barman et al. (24) pointed out that outpatient care was more impoverishing than inpatient care in urban and rural areas alike.

Government subsidies per inpatient and outpatient day were estimated INR 795 (Table 4) and INR 103 (Table 5), respectively. Subsidies per inpatient day for all types of diseases were higher than outpatient days. In 2004, Government provided a total subsidy for health care of INR 335 billion, out of which INR 294 billion (Table 4) was for inpatient treatment and INR 41 billion (Table 5) for outpatient treatment in 2004. The majority share of the total government subsidy was utilized for treatment of non-communicable diseases.

**Table 4:** **Government subsidy per inpatient day and total subsidy for inpatient care by type of disease, 2004**.

	Government subsidy per inpatient day (INR)					Total subsidy in government (billion INR)				
	0–34	35–44	45–64	65+	Total	0–34	35–44	45–64	65+	Total
CD	548	1,154	770	896	737	31.6	26.2	25.8	7.3	90.0
NCD	655	1,072	1,011	937	892	27.2	29.4	46.0	18.3	119.5
Others	644	768	771	699	706	32.8	20.2	19.0	5.0	77.0
Total	621	985	896	872	795	94.9	77.0	93.3	30.3	294.1

*Source: authors’ own calculation from NSS 60th round unit-level data*.

**Table 5:** **Government subsidy per outpatient visit and total subsidy for outpatient care by type of disease, 2004**.

	Government subsidy per outpatient day (INR)					Outpatient visit in government (million)					Total subsidy for outpatient care in government (INR billion)				
	0–34	35–44	45–64	65 +	Total	0–34	35–44	45–64	65+	Total	0–34	35–44	45–64	65+	Total
CD	63.86	67.75	178.16	135.87	82.74	88.7	13.49	28.97	9.44	140.74	5.66	0.91	5.16	1.28	13.02
NCD	97.29	167.8	133.78	136.22	129.81	40.7	22.33	52.64	49.18	164.12	3.96	3.75	7.04	6.7	21.45
Others	141.4	−73.31	146.86	17.68	91.59	38.9	12.78	15.45	11.75	78.72	5.5	−0.94	2.27	0.21	7.04
Total	86.75	70.63	146.84	118.51	102.45	168.3	48.6	97.06	70.38	383.58	14.6	3.43	14.25	8.34	40.63

*Source: National Sample Survey 60 Round, 25.0 sub-round, 2004*.

### Health-care Spending on Communicable and Non-Communicable Diseases as a Share of Gross Domestic Product

In 2004, 87% of the total health-care spending in India (approximately INR 100,000 crores) was out-of-pocket spending. India spent 3.35% of the GDP, and out-of-pocket spending on health accounted 2.94% of it. Half of the out-of-pocket spending on health care was on account of the treatment of non-communicable diseases and 30% was due to communicable diseases during 2004.

### Health-care Expenditure on Communicable Diseases and Non-Communicable Diseases in India: A Futuristic Vision

The health-care expenditure on the communicable diseases was expected to grow rather slowly than non-communicable diseases. Health-care expenditure on communicable diseases in government facilities may rise by about 1.7 times by 2051 than its corresponding figure in 2006 (Table 6), whereas expenditure on non-communicable diseases in government facilities may increase in a much faster rate by 2.5 times within the same time frame. In the private health facilities, health-care expenditure on the communicable diseases are expected to increase from INR 26,243 crores to INR 42,222 crores and on non-communicable diseases from INR 45,735 crores to 114,413 crores during the same duration. The rise in health-care expenditure due to communicable diseases is relatively higher in the first part of the projection period (2006–26) compared to the second part (2026–2051).

**Table 6:** **Projected annual health-care cost (INR Crores) in government and private facilities for communicable and non-communicable diseases in India**.

	2006	2026	2051
**Communicable diseases**			
Government	4,352	5,877	7,364
Private	26,243	34,634	42,222
**Non-communicable diseases**			
Government	8,424	13,294	20,722
Private	45,734	72,535	114,413

*Source: authors’ own calculation from NSSO 60th round unit-level data*.

## Discussion

The study provides some insights into the upcoming health requirements in Indian health system. The age profile of the morbidity indicates a rising risk of non-communicable diseases as the population ages through demographic transition (2, 16, 25, 26). In spite of a higher cost of treatment, people prefer to visit private facilities than the public. In India, the private facilities are vast and diverse – ranging from a person from local medical shop to a super specialty hospital catering to medical tourists from abroad (27). These facilities are available even in small remote places and offers services as per the convenience of people. The cost of treatment in these private facilities also varies to a great extent. People frequently prefer to go to a nearby private facility for short term diseases, which requires outpatient visit. Choice among various private health practitioners arises when the disease is more severe and requires long-term care.

Though government provides subsidized services for public health facilities, people utilize these facilities mainly for inpatient care. The lower level government facilities such as the sub-centers and the primary health centers are not well equipped to serve for severe illnesses. The higher level and specialized government health facilities are often concentrated in urban places, and travel to these places requires higher level resources and often associated with wage loss (27). India, in the past decade, has introduced the Rashtriya Swasthya Bima Yojana, a health insurance scheme to cover the inpatient care expenses of the poor. It has also invested on the National Rural Health Mission in 2005, which provides budget support to expand and improve free primary care in public clinics. While these initiatives ensure government’s continued effort to improve health-care services and protect the poor from health related expenses, these barely promise on the step toward combating non-communicable disease risks.

## Conclusion

The non-communicable diseases require attention at a very early stage and once developed, medication continues throughout lifetime. Neglecting these diseases incapacitates a person and may be even life threatening. Ghaffar et al. (16) attributed the environmental factors as the major determinants of these health conditions, whereas they noted sedentary life style, extreme poverty, and inadequate health system as the obstacle to tackle the challenge of non-communicable diseases in South Asia.

If India wishes to reap the demographic dividend out of its favorable age-structural transition, it needs to focus on the health of the youth. Creating an enabling atmosphere for the youth to lead a healthy life style, create awareness about the ill health effects of smoking and drinking, regular health check-ups, and good dietary intake, are the some among the possible ways to avoid health risks from non-communicable diseases. In a nutshell, investing on youth health may result into a higher return in future.

## Limitations

We acknowledge the possible various limitation of the study. The main limitation stands as the prevalence of morbidity is based on the reported ailments, which is subject to the knowledge and awareness of a particular morbidity and may differ from estimate-based clinical data. The study produces the shift in disease share on the basis of pure demographic profile, which certainly sets a basic level of alarm to Indian health system, but inadequate in long run. A longitudinal study with clinical investigation will help a more detailed projection. However, we have tried to pinpoint the short fall of the Indian health system to tackle the future health problem, which will come along with the ongoing demographic transition.

## Author Contributions

Both the authors have made significant contributions at various levels of preparation of the manuscript. While Dr. DB has carried out the research, Prof. PA has given his immense input in shaping the paper idea.

## Conflict of Interest Statement

The authors declare that the research was conducted in the absence of any commercial or financial relationships that could be construed as a potential conflict of interest.

## Footnotes

^1^BRICS refers to the association of five major emerging national economies namely Brazil, Russia, India, China, and South Africa.

## Appendix

**Table A1:** **Classification of communicable and non-communicable diseases**.

Communicable disease	Diarrhea/dysentery, gastritis/gastric or peptic ulcer, worm infestation, amebiasis, hepatitis/jaundice, tuberculosis, diseases of kidney/urinary system, conjunctivitis, sexually transmitted diseases, malaria, eruptive, mumps, diphtheria, whooping cough, fever of unknown origin, tetanus, and filariasis/elephantiasis
Non-communicable disease	Heart disease, hypertension, respiratory including ear/nose/throat ailments, bronchial asthma, disorders of joints and bones, prostatic disorders, gynecological disorders, neurological disorders, psychiatric disorders, glaucoma, cataract, diseases of skin, goiter, diabetes mellitus, anemia, locomotor, visual including blindness (excluding cataract), speech, hearing, and diseases of mouth/teeth/gum, cancer, and other tumors
Others	Under-nutrition, accidents/injuries/burns/fractures/poisoning, other diagnosed ailments, and other undiagnosed ailments
